# Heat induction in two-dimensional graphene–Fe_3_O_4_ nanohybrids for magnetic hyperthermia applications with artificial neural network modeling

**DOI:** 10.1039/d1ra03428f

**Published:** 2021-06-18

**Authors:** M. S. Dar, Khush Bakhat Akram, Ayesha Sohail, Fatima Arif, Fatemeh Zabihi, Shengyuan Yang, Shamsa Munir, Meifang Zhu, M. Abid, Muhammad Nauman

**Affiliations:** State Key Laboratory for Modification of Chemical Fibers and Polymer Materials, International Joint Laboratory for Advanced Fiber and Low-dimension Materials, College of Materials Science and Engineering, Donghua University Shanghai 201620 P. R. China shamraiz@mail.dhu.edu.cn zmf@dhu.edu.cn; Centre for Advanced Electronics and Photovoltaic Engineering (CAEPE), International Islamic University Islamabad Pakistan shamraiz@mail.dhu.edu.cn; School of Applied Sciences & Humanities, National University of Technology (NUTECH) Main IJP Road, Sector I-12 Islamabad Pakistan; Department of Mathematics, COMSATS University Islamabad Lahore Campus 54000 Pakistan; Department of Mechanical Engineering, COMSATS University Islamabad (Wah Campus) G.T. Road Wah Cantt Pakistan; Thermodynamics of Quantum Materials at the Microscale Laboratory, Institute of Science and Technology (IST) Austria

## Abstract

We report the synthesis and characterization of graphene functionalized with iron (Fe^3+^) oxide (G-Fe_3_O_4_) nanohybrids for radio-frequency magnetic hyperthermia application. We adopted the wet chemical procedure, using various contents of Fe_3_O_4_ (magnetite) from 0–100% for making two-dimensional graphene–Fe_3_O_4_ nanohybrids. The homogeneous dispersal of Fe_3_O_4_ nanoparticles decorated on the graphene surface combined with their biocompatibility and high thermal conductivity make them an excellent material for magnetic hyperthermia. The morphological and magnetic properties of the nanohybrids were studied using scanning electron microscopy (SEM) and a vibrating sample magnetometer (VSM), respectively. The smart magnetic platforms were exposed to an alternating current (AC) magnetic field of 633 kHz and of strength 9.1 mT for studying their hyperthermic performance. The localized antitumor effects were investigated with artificial neural network modeling. A neural net time-series model was developed for the assessment of the best nanohybrid composition to serve the purpose with an accuracy close to 100%. Six Nonlinear Autoregressive with External Input (NARX) models were obtained, one for each of the components. The assessment of the accuracy of the predicted results has been done on the basis of Mean Squared Error (MSE). The highest Mean Squared Error value was obtained for the nanohybrid containing 45% magnetite and 55% graphene (F_45_G_55_) in the training phase *i.e.*, 0.44703, which is where the model achieved optimal results after 71 epochs. The F_45_G_55_ nanohybrid was found to be the best for hyperthermia applications in low dosage with the highest specific absorption rate (SAR) and mean squared error values.

## Abstract 2

Synthesis of Fe_3_O_4_–graphene (FG) nanohybrids and magnetothermal measurements of F_*x*_G_100–*x*_ (*x* = 0, 25, 45, 65, 75, 85, 100) nanohybrids (25 mg each) at a 633 kHz alternating magnetic field of strength 9.1 mT.

## Graphical abstract

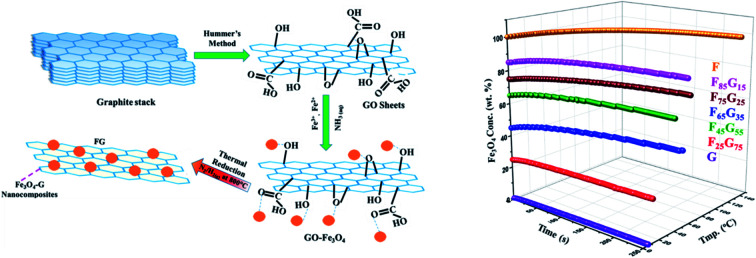

## 1. Introduction

Cancer treatment with high accuracy is a major concern of the medical community. For instance, hyperthermia or heat mediated therapy has today become of great significance utilizing energy absorbing nanoparticles.^1,2^ Cancer is one of the biggest challenges to humanity. According to the factsheet of February 2018 issued by the world health organization (WHO), cancer is the cause of almost 8.8 million deaths annually.^3^ It is imperative to develop an effective and accurate treatment method for this malignant disease. The typical therapies in-practice for cancer treatment include radiation therapy, chemotherapy, and surgery. However, apart from being painful, these therapies lead to several side effects such as damage to healthy tissues, fatigue, alopecia and multidrug resistance (MDR). Magnetic hyperthermia (HT) is an alternative and promising non-invasive approach for cancer treatment, where magnetic thermoseeds are injected directly into the tumor area of the patient.^4^ The cancerous area, containing the implanted thermoseeds, is heated to an elevated temperature through magnetic nanoparticles (MNPs). The heating phenomena is mainly induced in the magnetic nanoparticles under alternating magnetic field due to hysteresis, Néel and Brownian relaxation losses. Brown relaxation losses occurs when the nanoparticle rotate in the fluid and produce heat *via* a fraction mechanism in the aqueous medium. As there is a reduced blood flow in the tumor area, containing disorganized blood vessels, the heat dissipation to the surrounding area is limited. Therefore, cancerous cells are more susceptible for apoptosis at relatively mild heating up to ∼42 °C as compared to healthy cells. It has been reported that a temperature range of 41.8–44 °C provides the most suitable conditions for entire body hyperthermia.^5^ This is due to the leaky vasculature of the cancer cells that obstructs dissipation of thermal energy from them, as compared to the well-ordered blood vessels and nerves connected to healthy cells that can stand against heat by a more efficient heat dissipation, and a greater excretion of the heat shock proteins.^6,7^ The unique feature of nanoparticles to act selectively on the tumor cells sparing the healthy cells makes this therapeutic technique much more accurate as compared to the conventional methods.^8,9^

Ferrites such as Fe_3_O_4_ remained a hot contestant in hyperthermia applications due to their biocompatibility, strong intrinsic magnetic properties and their use in bio-medical applications. The use of these materials in magnetic resonance imaging (MRI) contrast enhancement, magnetic hyperthermia cancer therapy, and targeted drug delivery is a manifestation of their biocompatibility.^10–15^ However, the bare pristine Fe_3_O_4_ nanoparticles possess strong anisotropic dipolar interactions and high magnetization that result in agglomeration and precipitation. Due to these factors, their colloidal solubility is lost, and their activity is reduced. Therefore, it is challenging to incorporate Fe_3_O_4_ nanoparticles in both *in vitro* and *in vivo* experiments. To prevent their agglomeration and precipitation, we have developed a support of reduced graphene oxide sheets, for making Fe_3_O_4_ nanoparticles immobilized.

Graphene is a single-atomic-thin planar sheet of sp^2^ bonded carbon atoms. The introduction of the exfoliation technique in 2006 for producing single-layer graphene,^16^ has gained tremendous attention from application perspective. Large thermal conductivity (*κ* ∼ 5.3 × 10^3^ W m^−1^),^17,18^ high flexibility and strength (elastic stiffnesses ∼340 N m^−1^, Young's modulus ∼ 1.0 TPa, and breaking strength ∼42 N m^−1^),^18–20^ and excellent biocompatibility of graphene lead to remarkable properties for the development of prototype devices for biological applications such as miniaturized single fat-cell glucose sensors,^21^ graphene-based single-bacterium bio-device, label-free DNA sensor, and bacterial DNA/protein.^22^ Controlled tunability of the fabrication and functionalization of graphene is required, to achieve a remarkable performance from graphene-based devices, making it an important topic of contemporary research.

Thus far different techniques have been developed to produce graphene stacks of varying thicknesses, a post-functionalization to make hybrids of graphene with other materials. Morphologies and properties of free standing nano-islands of graphene in layered hybrid systems have been found to be closely related to their growth strategies.^23–25^ Among all, Hummer's method is the most famous and reliable for economical large-scale production of graphene. The Graphene Oxide (GO) obtained by Hummer's method is hydrophilic owing to numerous attached functional groups (hydroxyl, carbonyl, epoxide, carboxyl^26^), making the graphene easy to functionalize with other species. Cong *et al.* reported the synthesis of hydrazine reduced GO sheets (prepared by modified Hummer's method), that were post decorated with Fe_3_O_4_ nanoparticles. These Fe_3_O_4_ functionalized graphene sheets could be used as a magnetic resonance contrast. Jing Su *et al.*^27^ prepared graphene–Fe_3_O_4_ nanohybrids *via* a hydrothermal approach. These materials exhibit superparamagnetic properties for biocompatible controlled drug delivery.

Although Hummer's method provides an efficient approach for the introduction of new species to a 2D graphene oxide system in the form of hybrids however, reduction of GO is an important stage for a reliable functionalization of defect free graphene. In this work, we adopted thermal reduction method for producing graphene and Fe_3_O_4_–graphene nanohybrids. The synthesized nanohybrid materials are applied to investigate magnetic hyperthermia and their functionality is compared with that of pristine Fe_3_O_4_ and reduced graphene oxide. Large specific heat capacity of graphene and the charge transfer effect between graphene sheets and immobilized magnetic Fe_3_O_4_ nanoparticles enhanced the magnetic hyperthermia effect. The best thermoseed agent was determined by calculating the specific absorption rate (SAR).

Artificial intelligence (AI) has been dominant in health care and medical sciences since the advent of current century. Researchers and futurists have concluded that the collaboration of this technology with the doctors can make wonders and that there is still a long way for scientists to go to unveil the extraordinary potential of AI to transform health care into a much more modern and efficient medical care system. Keeping this in view, we have also utilized AI to produce improved nanoparticles for hyperthermia treatment.

We have used a deep learning algorithm for the time series modeling of hyperthermia data. Deep learning is a subset of machine learning which is a subfield of a bigger domain *i.e.*, AI. Deep learning consists of artificial neural networks (ANN) based algorithms. ANNs have been used by scientists to analyse time series data for prediction and forecasting.^28,29^

We have developed a system of six artificial neural networks to assess the best nanoparticle. ANNs with exogenous input have this capability to extract information from the past values in the data and process it to learn and then predict step ahead values which gives us an insight of the performance of each particle. However, the algorithm requires as much data as possible for improved predictions.

## 2. Materials and methods

For synthesis of Fe_3_O_4_–graphene nanohybrids, high quality expandable graphite powder of mean size 25 μm was purchased from Aldrich (purity 99.99%), FeCl_3_·6H_2_O from Riedel-de Haen (purity 99%), FeCl_2_·4H_2_O (purity 99.8%) and HCl from Merck, KMnO_4_ from BDH (purity 99%), 32% NH_3_ solution and high grade H_2_SO_4_ and H_2_O_2_ (30 wt%) were purchased from Panreac. All reactions were carried out using deionized (DI) water.

### 2.1. Synthesis of graphene oxide (GO)

GO was synthesized using graphite powder as a starting material by modified Hummer's method.^30^ 5 g graphite powder was added in 125 mL H_2_SO_4_ (99.99% assay) in a flat-bottomed flask at 0 °C followed by vigorous stirring to avoid agglomeration. Once the powder was well dispersed, 15 g KMnO_4_ was added to the mixture slowly, at a low temperature, below 15 °C. Gradually the mixture was brought to room temperature. After the reaction, mixture became pasty and turned light brown in color. 150 mL of DI water was added slowly to the mixture to dilute it, after which 17 mL H_2_O_2_ (30 wt%) was added, that changed the mixture color to yellow. Finally, the mixture was washed with 1 : 10 HCl (1 M) solution to remove residual ions. Grey colored GO powder was obtained after drying the solution in oil bath with continuous stirring (10 rpm) for 8 hours at room temperature (25 °C).

### 2.2. Synthesis of magnetite–graphene oxide (Fe_3_O_4_–GO) compositions

Magnetite–graphene oxide F_*x*_G_100–*x*_ compositions have been synthesized, where *x* (= 0, 25, 45, 65, 75, 85, 100) refers to the weight percentage of magnetite in the nanohybrid. Note the composition with *x* = 0 specifies pure graphene and *x* = 100 specifies pure magnetite. Specific amounts of FeCl_3_·6H_2_O and FeCl_2_·4H_2_O and graphene oxide (GO) were weighed for each composition. Stoichiometric quantities of FeCl_3_·6H_2_O and FeCl_2_·4H_2_O were dissolved in 25 mL de-ionized (DI) water to obtain their 0.04 and 0.02 molar aqueous solutions, respectively. The required amount of GO was dispersed in DI water (250 mL H_2_O for 0.9 g GO) for each composition. In order to transform the attached carboxylic acid groups to carboxylate anions, the dispersion was sonicated for 1 h. Then, 0.02 mole of FeCl_2_·4H_2_O and 0.04 mol of FeCl_3_·6H_2_O were dissolved in DI water and added dropwise to the GO solution at room temperature with vigorous stirring. On completion of ion exchange reaction, 32% NH_3_ solution was added drop by drop until the pH of solution became 10 that is required for the formation of Fe_3_O_4_ NPs.^31^ All the compositions were dried in oil bath with continuous stirring (10 rpm) for 8 hours at room temperature (25 °C).

### 2.3. Thermal reduction

**Fig. 1:**
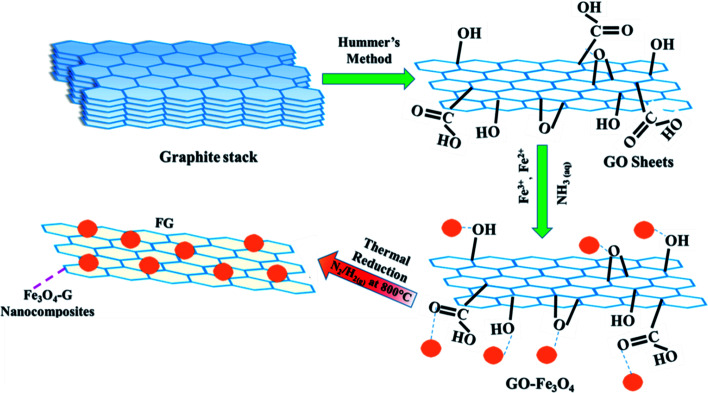
Schematic diagram for synthesis of Fe_3_O_4_–graphene nanohybrids: a commercially available graphite stack was converted to GO using Hummer's method. Iron-II (Fe^2+^) and iron-III (Fe^3+^) ions for Fe_3_O_4_ NPs synthesis were introduced on GO templates with functional groups attached. Later GO was reduced thermally for exfoliation purpose.

For the fabrication magnetite–graphene (FG) nanohybrids, Fe_3_O_4_–GO compositions in dried powder forms were thermally reduced in a quartz tube furnace for 1 h at 800 °C (heating rate 10 °C min^−1^) at 300 cm^3^ flow rate of forming gas (N_2_/H_2_, 95%/5%). The samples were named according to the weight % ratio in the compositional formula F_*x*_G_100–*x*_ (*x* = 0, 25, 45, 65, 75, 85, 100) as G, F_25_G_75_, F_45_G_55_, F_65_G_35_, F_75_G_25_, F_85_G_15_ and F, respectively. For example, F_45_G_55_ refers to the nanohybrid containing 45 wt% magnetite and 55 wt% graphene. Schematic diagram for synthesis of FG nanohybrids is shown in Fig. 1.

### 2.4. Synthesis of magnetite (Fe_3_O_4_) nanoparticles

Magnetite nanoparticles were prepared by co-precipitation^31^ method. Fe_3_O_4_ NPs were obtained on reacting iron-II (Fe^2+^) and iron-III (Fe^3+^) ions in an ammonia solution (pH 10) in the molar ratio 1 : 2 respectively, at room temperature by stirring the solution for 30 min at 4000 rpm, following the reaction in eqn (1)

1 2FeCl_3_ + FeCl_2_ + 8NH_3_ + 4H_2_O → Fe_3_O_4_ + 8NH_4_Cl

The solution was washed several times at room temperature by stirring the dispersion for 30 min at 3000 rpm, to remove the unwanted ions and until it attained a pH of 7. Magnetite nanoparticles were centrifuged from the solution and dried at room temperature.

## 3. Artificial neural network (ANN) modeling

Machine learning is a complementary tool for analyzing an extract hidden trends in time series. Due to non-linear behavior of our time series, Artificial neural network for mathematical modeling has been chosen as they possess the ability to carry out non-linear mappings. This technique has been of considerable usability in the field of time series forecasting.^15,32,33^ Furthermore, several models based on nonlinear autoregressive structure have been proposed.^34–36^ We have developed a neural net time-series model using Neural Net Time Series app on MATLAB R2018b. It takes one or more time series for prediction; however, our time-series difficulty is to make a prediction by utilizing the *l* past values of a time series which is being predicted (*y*(*t*)), and another time-series (*x*(*t*)), in our case the 100% magnetite particle. In this basis, we have used Nonlinear Autoregressive with External Input (NARX) neural network. NARX is a recurrent network capable to model dynamic systems,^2^ it is not only able to predict output value which is regressed on the previous values but is also used for nonlinear filtering (eqn (2)):

2 *y*(*t*) = *g*(*x*(*t* − 1), …, *x*(*t* − *l*), *y*(*t* − 1), …, *y*(*t* − *l*))

**Fig. 2:**
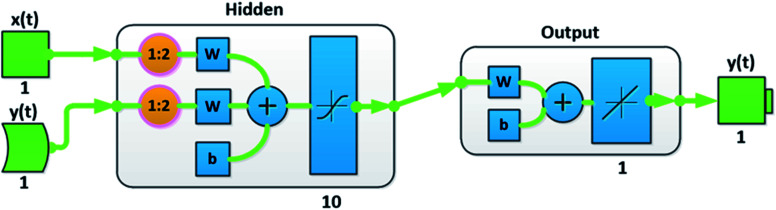
Neural network architecture.

Data is fed into the three layered (namely input, hidden and output layers) network consisting of 10 hidden neurons and two-time delays (Fig. 2). Size of the hidden layer was chosen carefully after experimenting with other combinations and the time delays were added to incorporate the dynamic of the input dataset. The division of target time steps has been done as: 70% for training, 15% for validation and 15% for testing. Furthermore, Bayesian Regularization (BR) back propagation algorithm is used to train the network. Regularization is required to solve the overfitting problem. To confront the performance decline, generalization errors regularization has been used skilfully. A number of techniques are available to serve the purpose.^37^ However, our choice of Bayesian regularization is influenced by the involvement of Bayesian theorem which incorporates prior data along with maximum likelihood function to give posterior distribution.^38^

BR happens to be a better choice for quantitative researches, due to its potential to unveil complex data patterns and interrelations.^39^ Adopting a probabilistic approach towards machine learning is the most reliable strategy to solve a problem like future prediction which is based on uncertainty.^40^ It may take a little longer but yields finer results and has emerged to be the most robust and vigorous one in comparison to the standard back propagation NNs. Regularization itself is meant to overcome the overfitting issue, hence the models trained using BR are difficult to overtrain and overfit.^41^ It utilizes posterior probability which involves utilization of Bayesian theorem for parametric optimization and updating the knowledge from prior to posterior.^42^ Bayesian inference is a highly commendable approach for statistical analysis of stochastic processes.^43^

The training process continues till the optimal result is achieved and is terminated as the generalization stops improving, the model can be trained for the maximum number of 1000 epochs.

After training, the assessment of the accuracy of the predicted results is carried out on the basis of Mean Squared Error (MSE) eqn (3), which is the average of squared difference of output and target values:3
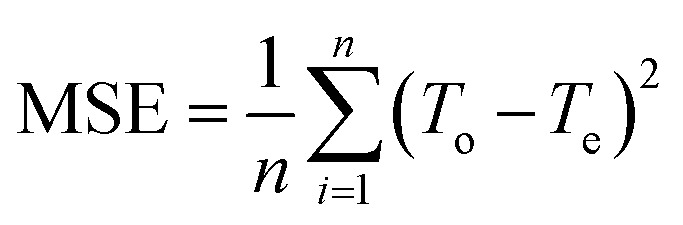
where *T*_o_ are the observed values and *T*_e_ are the estimated ones. Moreover, regression (*R*) values have been calculated to see the correlation between output and target. Values of *R* close to 1 are considered to be optimal, it indicates close relationship between output and target.

## 4. Results and discussion

**Fig. 3:**
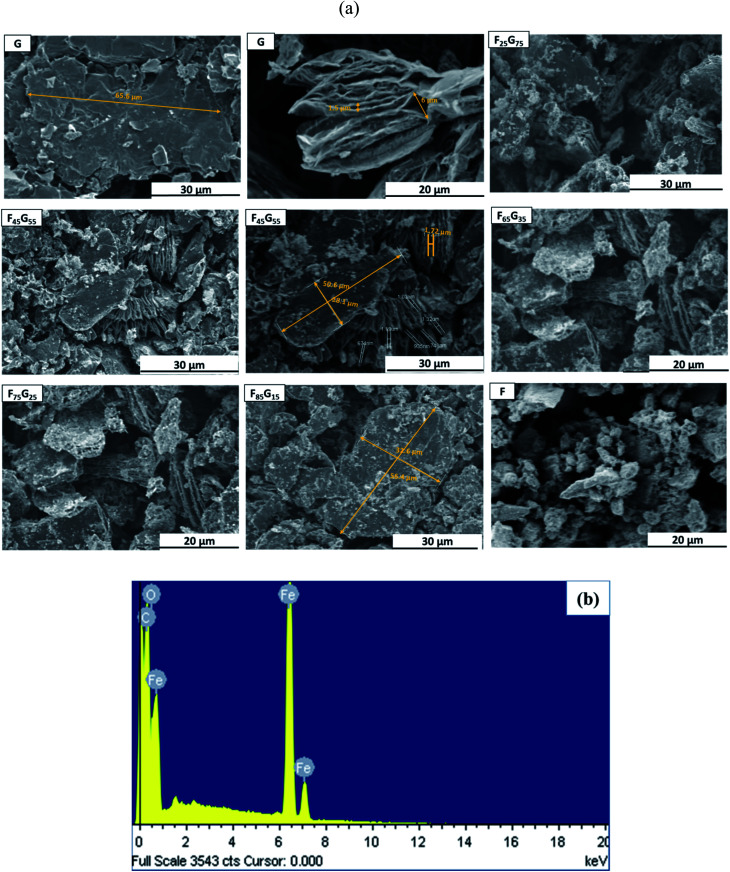
(a) Scanning electron microscopy (SEM) images of thermally reduced graphene flakes (G), Fe_3_O_4_–graphene (FG) nanohybrids and pure Fe_3_O_4_ (F) nanoparticles. Yellow lines are guide to eye for the size of graphene flakes. (b) Energy dispersive X-ray (EDX) spectra of sample F_75_G_25_.

JEOL (Japan) JSM-6400F field emission scanning electron microscopy (FE-SEM) equipped with electron diffraction microscopy (EDX) was used for morphological and elemental analysis of Functionalized Graphene (FG) nanohybrids as presented in Fig. 3(a) and (b) respectively. The first two images on the top left side of Fig. 3(a) labelled as G show multilayer graphene flakes with a micron scale length *i.e.*, 65.6 μm for sample G. It is evidenced that for low Fe_3_O_4_ samples (up to 45%), Fe_3_O_4_ nanoparticles are evenly distributed within the graphene matrix with a very little degree of agglomeration. However, with increasing the content of Fe_3_O_4_, further agglomeration is observed. It can be concluded that, the average size of Fe_3_O_4_ nanoparticles significantly varies with the content of Fe_3_O_4_ with nano scale length. The image in Fig. 3(a) labeled as F, (for pure magnetite) shows agglomerated Fe_3_O_4_ nanoparticles with non-uniform size distribution due to magnetic anisotropic interactions.

To confirm the presence of the Fe_3_O_4_ on the graphene surface sample F_75_G_25_ was randomly selected for energy dispersive X-ray spectroscopy (EDX) as shown in Fig. 3(b). From EDX results the presence of Fe, O and C, confirms that the Fe_3_O_4_ nanoparticles are distributed between the layers of the graphene sheets, which lead to the formation of Fe_3_O_4_–graphene nanohybrids.

**Fig. 4:**
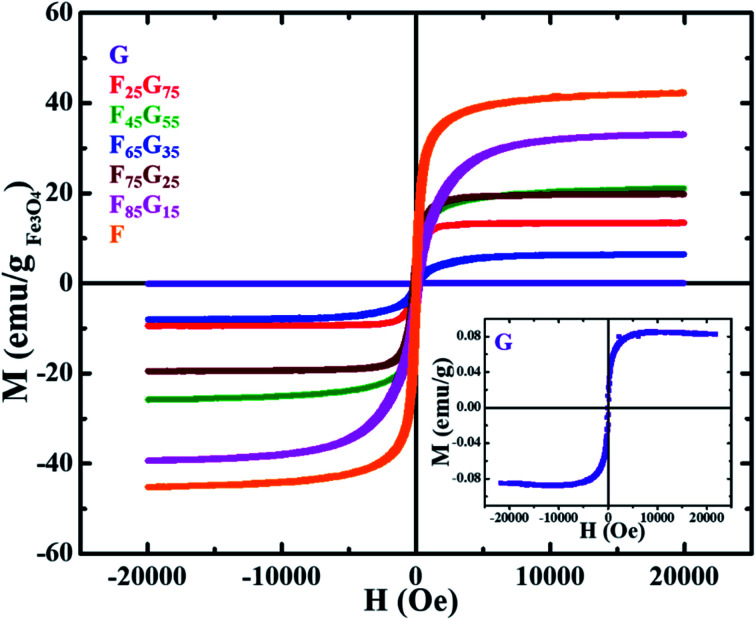
Room temperature magnetization hysteresis loops of thermally reduced Fe_3_O_4_–graphene (FG) nanohybrid samples normalized by sample mass and mass of the magnetic component (Fe_3_O_4_). The inset shows the M(*H*) loop of thermally reduced pure graphene (G) measured at room temperature.

Pristine graphene, Fe_3_O_4_ and FG samples were exposed to DC magnetic field, up to 20 kOe, in Lake Shore 7404 (US) vibrating sample magnetometer (VSM), at room temperature. The field dependent M(*H*) curves of all the samples (Fig. 4) were normalized by the mass of the sample and mass of the magnetic component (Fe_3_O_4_) for a comparative study of their magnetic properties. The inset shows the M(*H*) loop of pure graphene. Remanence (*M*_r_) and coercivity (*H*_c_) values for all samples are shown in Table 1 below.

**Table 1:** Magnetic remanence (*M*_r_) and coercivity (*H*_c_) values of pure and nanohybrid samples

Compositions	*M*_r_ (±0.010) (emu g^−1^)	*H*_c_ (±0.01) (kOe)
G	0.019	70.641
F_25_G_75_	1.933	82.334
F_45_G_55_	5.916	52.140
F_65_G_35_	1.175	120.491
F_75_G_25_	6.215	125.919
F_85_G_15_	4.752	138.383
F	12.630	140.276

All the samples demonstrated ferromagnetic behavior. Table 1 shows that magnetic remanence (*M*_r_) and coercivity (*H*_c_) values of samples are not increasing monotonically with the Fe_3_O_4_ content. As seen in SEM images Fig. 3(a) with the increase in Fe_3_O_4_ content, further agglomeration is observed. It can be seen from combined analysis of SEM and VSM results that *H*_c_ and *M*_r_ values are mainly dependent on degree of agglomeration of Fe_3_O_4_ rather than its concentration. Moreover, the magnetic behavior of a magnetic component dispersed in a nonmagnetic reduced graphene oxide matrix depends not only on the quantity of the magnetic component but also on how it is dispersed within the matrix.^44^ The weak magnetization of graphene is due to the presence of local defect states and non-magnetic nature.^45^ Sample G attains a maximum saturation magnetization of 0.08 emu g^−1^. The measured saturation magnetization (*M*_S_) of pure Fe_3_O_4_ (sample-F) ∼42 emu g^−1^ is much lower than the value reported for bulk counterpart (85–95 emu g^−1^). This is due to the well-understood size dependence of magnetization in nanoparticles in which surface spin disorder can lead to a magnetically dead surface layer.^46^ In case of FG samples, the magnetic behavior (*M*_r_, *M*_s_ and *H*_c_) is not depending upon the magnetic component. For example, remanence and saturation magnetization of the F_45_G_55_ sample is comparable to that of F_75_G_25_*i.e.*, ∼6 emu g^−1^ and 20 emu g^−1^ respectively. This can be due to the intercalation-dependent magnetic interactions. As shown in the SEM images (Fig. 3), there is still a slight degree of agglomeration with the lower contents of magnetic element. Therefore, increasing content will lead to increasing intercalation and exfoliation that results in the frustration of magnetic moments and enhanced dipolar interactions. These all could lead to a decrease in magnetization, associated with an increase of magnetic content over 45%.

**Fig. 5:**
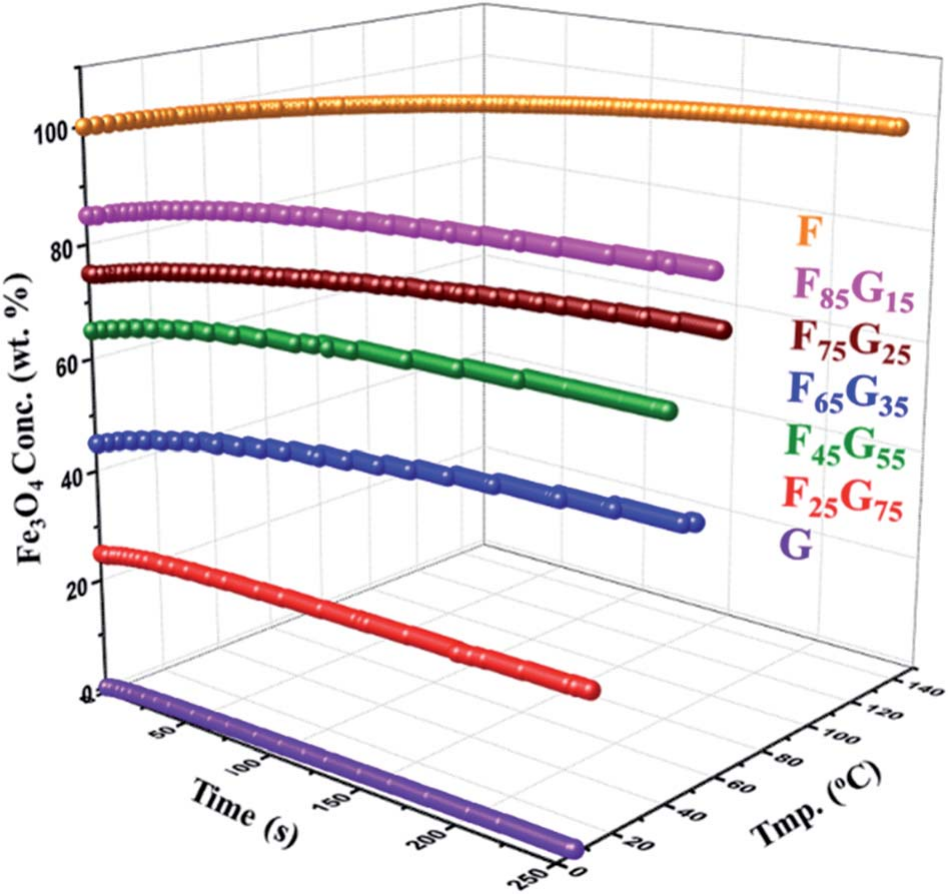
Hyperthermia measurements of FG nanohybrids (25 mg each) at 633 kHz alternating magnetic field of strength 9.1 mT.

Hyperthermia response of pure samples and FG nanohybrid (F_25_G_75_, F_45_G_55_, F_65_G_35_, F_75_G_25_, F_85_G_15_) was measured using NAN201003 MagneTherm (UK) induction heating unit for 25 mg of each sample powder exposed to a 633 kHz alternating magnetic field of strength 9.1 mT. A significant heating upshot was observed in all the synthesized nanohybrids (Fig. 5). There is a negligible heating in the pure graphene (sample-G) due to its weak magnetization. A considerable heating effect was detected, for all the FG nanohybrid, as well as the pure Fe_3_O_4_ samples. This trend can be assigned to Néel and hysteresis losses in single and multidomain particles respectively.^47,48^

Specific absorption rate (SAR) is the rate at which MNPs convert magnetic energy into heat. SAR is considered as a figure of merit in hyperthermia measurements. The specific absorption rate (SAR) of the samples have been calculated from the heating curves, using the following eqn (4).4
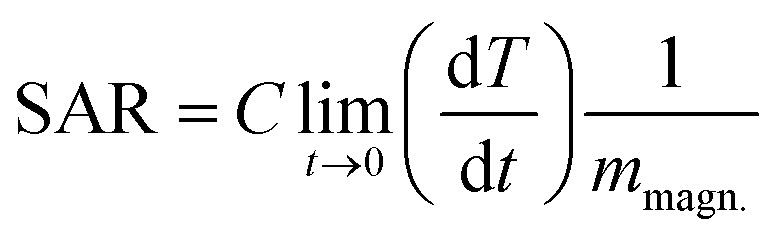
where, 
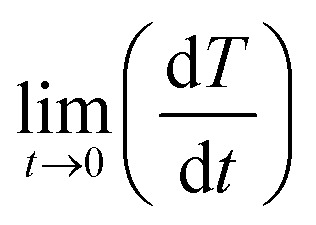
 is the initial heating rate, *C* is the heat capacity of nanohybrid sample and *m*_magn._ is the mass of magnetic component in the sample. The value of specific heat capacity (*C*) of each nanohybrid sample was determined by weight % contribution of Fe_3_O_4_ and graphene in the given composition. Specific heat capacity of Fe_3_O_4_ was taken as 0.937 J g^−1^ K^−1^ (ref. 49) and that of graphene was taken as 1.958 J g^−1^ K^−1^.^17^Table 2 shows specific heat capacities and SAR values of pure and nanohybrid samples.

**Table 2:** Specific heat capacities (*C*) and specific absorption rate (SAR) values of pure and nanohybrid samples

Compositions	*C* (±0.010) (J g^−1^ K^−1^)	SAR (±0.01) (W g^−1^)
G	1.958	0.00
F_25_G_75_	1.705	4.24
F_45_G_55_	1.501	6.45
F_65_G_35_	1.297	3.61
F_75_G_25_	1.195	1.78
F_85_G_15_	1.093	2.66
F	0.937	4.32

**Fig. 6:**
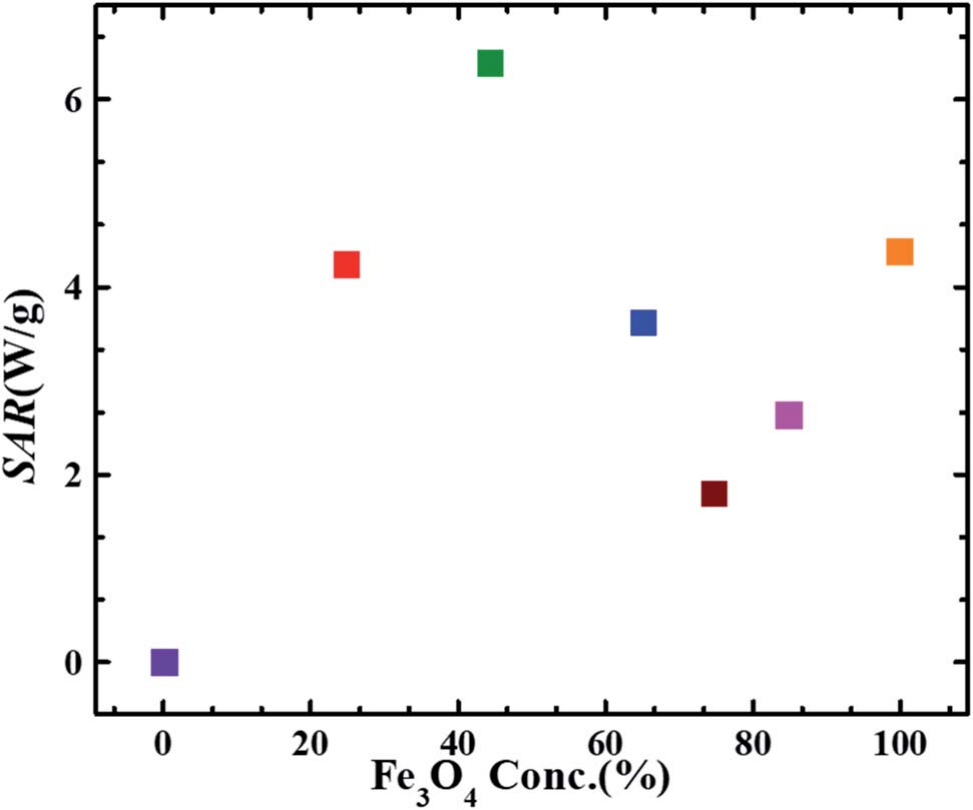
SAR values obtained at 633 kHz alternating magnetic field of strength 9.1 mT as a function of magnetite content in FG nanohybrid samples. (Error bars are within the symbol size).

Fig. 6 depicts the trend of the SAR values *vs.* magnetite content w.r.t wt% ratio in different FG nanohybrids. SAR value obtained for pure magnetite sample (F) is smaller than that reported by P. Burnham *et al. i.e.*∼5.813 W g^−1^ for dry magnetite.^50^ This difference is possibly due to the magnetic field strength (200 Oe), alternating frequency (282 kHz) and particle size (particle size = 15.3 nm) difference. Interestingly, the SAR value of F_45_G_55_ sample is ∼1.5 times larger and appx. same for F_25_G_75_ samples, as compared to that of the pure magnetite (F). Such an intriguing feature must be due to the large area matrix provided by the reduced graphene oxide sheets, and its heat capacity which is higher than that of pure magnetite. In details, the heat capacity of graphene is about twice that of magnetite, leading to higher SAR for lower content of Fe_3_O_4_. Additionally, the highest SAR obtained for sample F_45_G_55_*i.e.*, 6.45 W g^−1^, is due to an excellent exfoliation of graphene and most uniform intercalation of magnetite nanoparticles, as has been observed in SEM images. These behaviors also led to reduced frustration of moments for highest saturation magnetization (Fig. 4). Therefore, F_45_G_55_ sample can be considered as best candidate among all other nanohybrid compositions with highest SAR for hyperthermia applications in low dosage. Inductive heating property of graphene oxide–Fe_3_O_4_ nanoparticles hybrid in an AC magnetic field has been studied by Li-Zhong Bai *et al.* for localized hyperthermia applications^22^ however they have not calculated SAR values for analyzing the hyperthermia efficacy for their samples. Table 3 shows a comparison of the SAR values obtained for our best hybrid composition F_45_G_55_ and other most commonly reported agents for magnetic hyperthermia. The first row represents our hybrid composition with highest SAR among our composition and that of the other reported materials. This is an important result for hyperthermia applications of graphene based nanohybrids in low dosage.

**Table 3:** Comparison of the measured specific absorption rate (SAR) with reported literature

Compositions	SAR (±0.01) (W g^−1^)	Field H (Oe)	Frequency (kHz)	Ref.
F_45_G_55_	6.45	91	633	Current study
Fe_3_O_4_	5.80	200	282	51
Gd_5_Si_4_	3.70	171	327	52
Fe_2_O_3_	0.50	133	500	53
NiFe_2_O_4_	0.43	133	500	53
ZnFe_2_O_4_	0.07	133	500	53
CoFe_2_O_4_	0.04	133	500	53
CuFe_2_O_4_	0.27	133	500	53
La_0.8_Sr_0.2_MnO_3_	0.91	133	500	54

## 5. Modeling

Six NARX models are obtained, one for each of the components in Table 2. NARX is a nonlinear regression which uses both endogenous and exogenous inputs. The assessment of the accuracy of the predicted results has been done on the basis of Mean Squared Error (MSE) described in eqn (3). Moreover, regression (*R*) values have been calculated to see the correlation between output and target. Calculated values for all the compounds are given in the following Table 4:

**Table 4:** MSE and *R* values calculated for FG compositions

Composition	MSE	*R*
G	0.00380	0.99880
F_25_G_75_	0.02592	0.99774
F_45_G_55_	0.44703	0.99836
F_65_G_35_	0.21048	0.99852
F_75_G_25_	0.14962	0.99970
F_85_G_15_	0.23729	0.99924

**Fig. 7:**
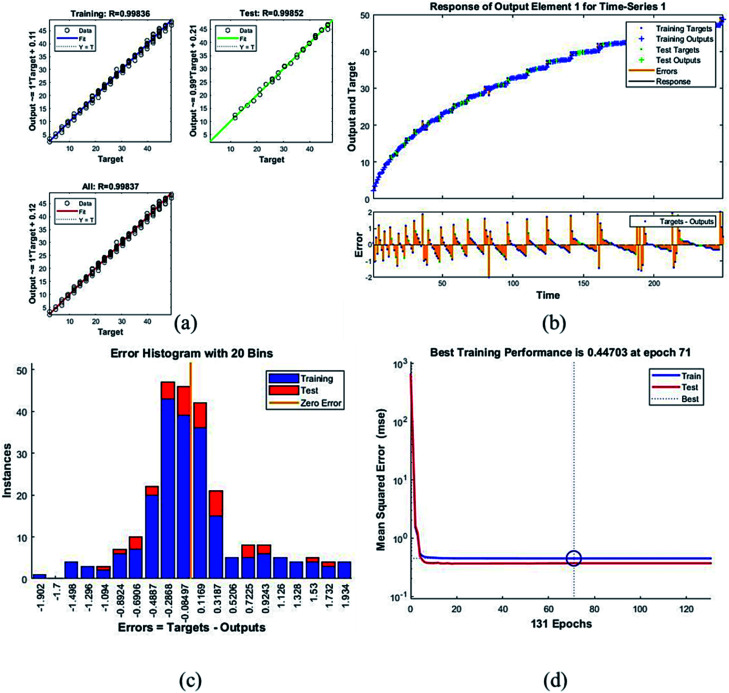
Plots for sample F_45_G_55_ (a) the correlation between the output and the targets. (b) A time series response curve with time instances on the *x*-axis and the predicted *versus* observed values on the *y*-axis. The other smaller graph in (b) is the magnified image of errors that were obtained in the time series response plot. (c) Error histogram with 20 bins (d) a performance plot MSE *versus* epoch count.

Table 4 presents the MSE and *R* values for the six composites. These values were obtained after completing the testing phase of the model. The model trained for the assessment of F_45_G_55_ performance in comparison to pure magnetite yielded highest MSE value. It turns out to be the most different one. Moreover, the *R* values depict the accuracy of the results obtained from the model which is also high for this composition. The plots obtained from the trained network for F_45_G_55_ are as following:

The regression/scatter plots on Fig. 7(a) depict the correlation between the output and the targets. Generally: output = *i* × target + *j*; where *i* is slope and *j* is the *y*-intercept. Here, we have three plots for different values of *j*. Each of them shows a meaningful correlation between target and output since the value of *R* is very close to 1, which is the ideal case. We have achieved maximum regression values for all the particles. Fig. 7(b) represents a time series response curve with time instances on the *x*-axis and the predicted *versus* observed values on the *y*-axis. The other smaller graph in Fig. 7(b) is the magnified image of errors that were obtained in the time series response plot. The targets are the correct data (that we gave in as input) whereas the outputs are the results obtained from the trained model *i.e.*, the predicted results. Our training and test outputs lie on the response line. Hence, the error is obtained by comparing the values from the response line to the ones that lie above or below the line. So, if the target value lies above the line, we obtain a positive error and, negative otherwise.

**Fig. 8:**
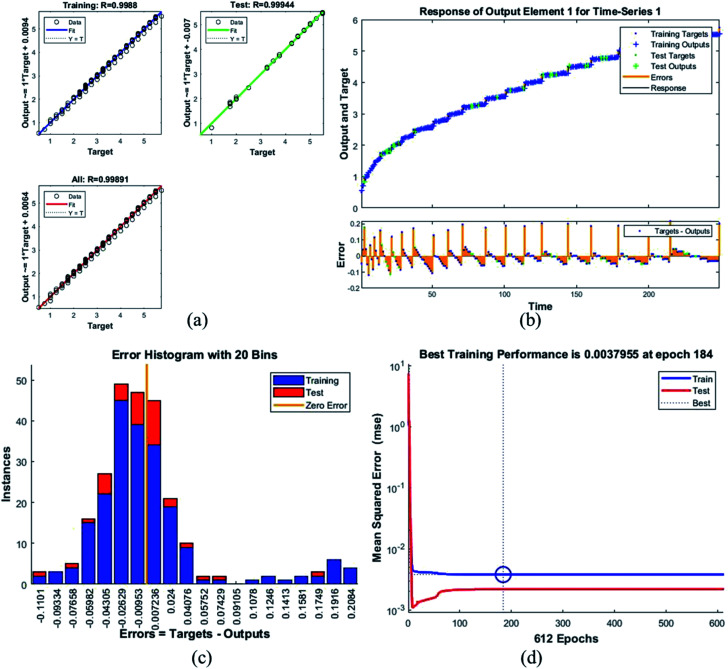
Plots for sample G (a) the correlation between the output and the targets. (b) A time series response curve with time instances on the *x*-axis and the predicted *versus* observed values on the *y*-axis. The other smaller graph in (b) is the magnified image of errors that were obtained in the time series response plot. (c) Error histogram with 20 bins (d) a performance plot MSE *versus* epoch count.

**Fig. 9:**
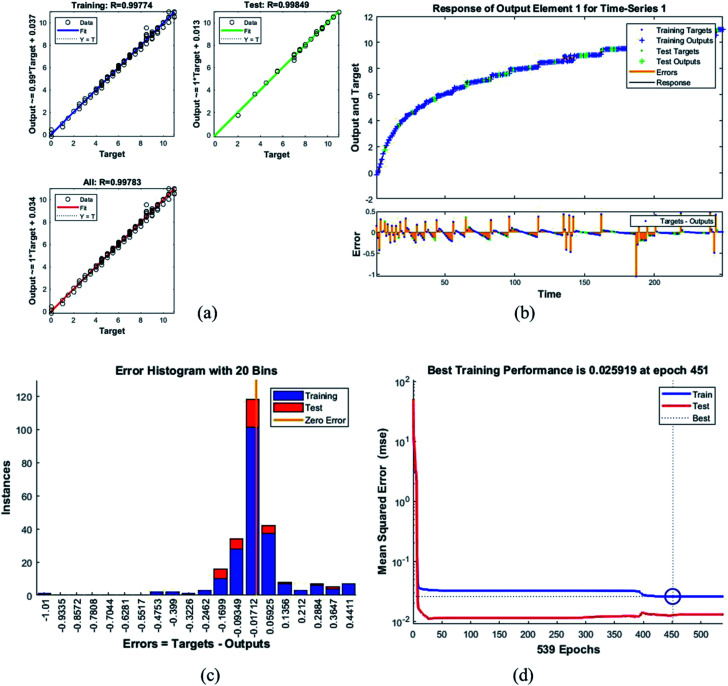
Plots for sample F_25_G_75_ (a) the correlation between the output and the targets. (b) A time series response curve with time instances on the *x*-axis and the predicted *versus* observed values on the *y*-axis. The other smaller graph in (b) is the magnified image of errors that were obtained in the time series response plot. (c) Error histogram with 20 bins (d) a performance plot MSE *versus* epoch count.

**Fig. 10:**
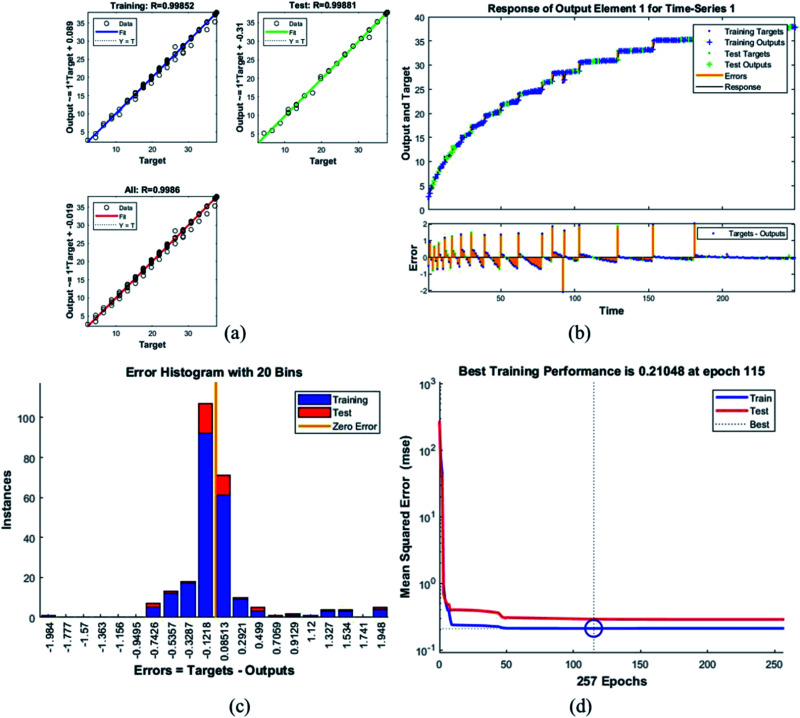
Plots for sample F_65_G_35_ (a) the correlation between the output and the targets. (b) A time series response curve with time instances on the *x*-axis and the predicted *versus* observed values on the *y*-axis. The other smaller graph in (b) is the magnified image of errors that were obtained in the time series response plot. (c) Error histogram with 20 bins (d) a performance plot MSE *versus* epoch count.

**Fig. 11:**
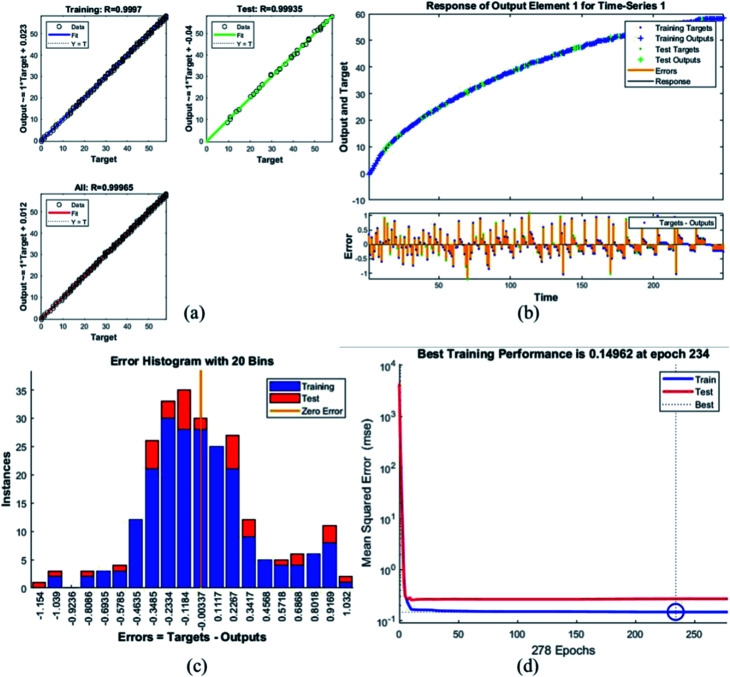
Plots for sample F_75_G_25_ (a) the correlation between the output and the targets. (b) A time series response curve with time instances on the *x*-axis and the predicted *versus* observed values on the *y*-axis. The other smaller graph in (b) is the magnified image of errors that were obtained in the time series response plot. (c) Error histogram with 20 bins (d) a performance plot MSE *versus* epoch count.

**Fig. 12:**
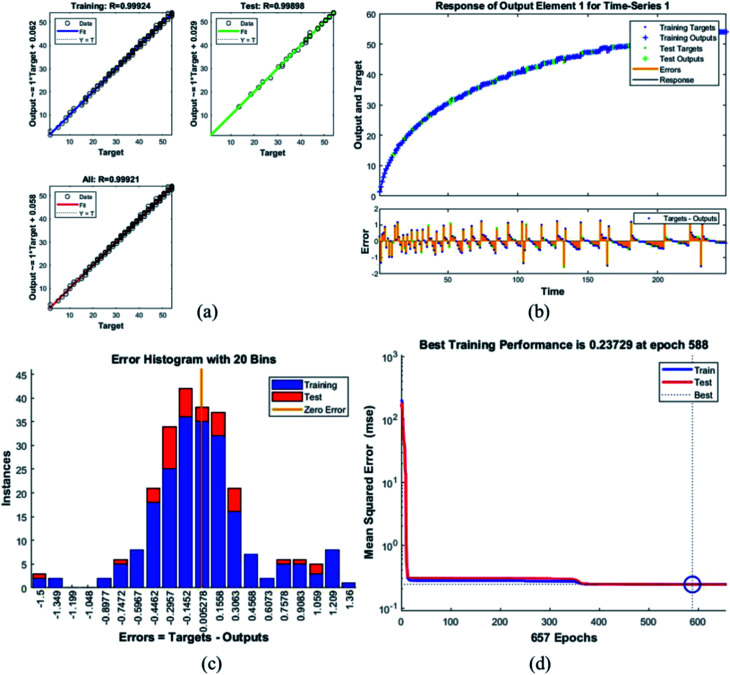
Plots for sample F_85_G_15_ (a) the correlation between the output and the targets. (b) A time series response curve with time instances on the *x*-axis and the predicted *versus* observed values on the *y*-axis. The other smaller graph in (b) is the magnified image of errors that were obtained in the time series response plot. (c) Error histogram with 20 bins (d) a performance plot MSE *versus* epoch count.

Error histogram with 20 bins (Fig. 7(c)) has number of instances on *y*-axis and errors on the *x*-axis. The maximum number of instances do not have zero error. This shows the predicted results are not very close to the target values, time series and performance plots further validate this interpretation. Fig. 7(d) is a performance plot MSE *versus* epoch count. As stated earlier, MSE is the difference between the observed and the simulated, therefore it should be lower. According to our motivation, the particle that obtained the highest error is the most favorite one, and for F_45_G_55_, MSE obtained in the training phase is 0.44703, where the model achieved optimal results after 71 epochs. However, it is seen that Bayesian regularization does not require validation, this is due to this fact that the sole purpose to perform validation check is to make sure either error increases or decreases while training and avoid overfitting in the testing phase, making Bayesian method encounters successfully while training the model. Most importantly, our experimental results also have shown that highest SAR value is obtained for sample F_45_G_55_ (Fig. 6). It means that, F_45_G_55_ composition can be an excellent candidate for cancer treatment *via* magnetic hyperthermia approach. The plots obtained from the trained network for other samples are shown in Fig. 8–12.

## 6. Conclusion

The goal of this work was to develop biocompatible two dimensional (2D) magnetic system of high thermal conductivity for the treatment of malignant tumors *via* the magnetic hyperthermia approach. 2D system of thermally reduced graphene functionalized with magnetite nanoparticles in different weight ratios (0–100%) were fabricated. All the compositions were characterized and analyzed morphologically and magnetically at microscopic level. Hyperthermia measurements showed a high specific absorption rate (SAR). Sample containing 45% magnetite and 55% graphene *i.e.*, F_45_G_55_ is found to have the largest SAR value of 6.45 W g^−1^ with saturation magnetization ∼20 emu g^−1^, that is ∼1.5 times greater than that of pure magnetite (4.32 W g^−1^) with saturation magnetization ∼42 emu g^−1^. The role of graphene is to reduce frustration of magnetic moments by monitoring the dipolar interactions with better exfoliation and low agglomeration of Fe_3_O_4_ nanoparticles. Moreover, large value of specific heat capacity of graphene contributes an increase in SAR of nanohybrids to even 1.5 times larger than that of the pure magnetite. Nonlinear Autoregressive with External Input (NARX) models are obtained for each of the nanohybrid composition. The accuracy of the predicted results has been evaluated on the basis of Mean Squared Error (MSE). The highest MSE value was obtained for the composition containing 45% magnetite and 55% graphene (F_45_G_55_) nanohybrid in the training phase *i.e.*, 0.44703 which is where the model achieved optimal results after 71 epochs. Hence, F_45_G_55_ sample was found the best nanohybrid with highest SAR and MSE values for hyperthermia applications in low dosage. With suitable surface functionalization, biocompatible Fe_3_O_4_-graphene nanohybrids can be useful candidates for localized magnetic hyperthermia applications.

## Conflicts of interest

The authors report no conflicts of interest.

## Supplementary Material
